# Affecting children’s knowledge about rational use of medicines using read-along videos of pictorial storybooks

**DOI:** 10.3389/fphar.2022.933546

**Published:** 2022-09-02

**Authors:** Syafi’ah Bakaruddin, Zakiah Mohd Noordin, Mahmathi Karuppannan

**Affiliations:** ^1^ Pejabat Kesihatan Wilayah Persekutuan Putrajaya, Putrajaya, Malaysia; ^2^ Department of Pharmacy Practice, Faculty of Pharmacy, Universiti Teknologi MARA Cawangan Selangor, Puncak Alam Campus, Bandar Puncak Alam, Malaysia

**Keywords:** rational use of medicine, pictorial storybook, Children, read-along video, pretest-posttest

## Abstract

Although efforts have been taken to educate the public about medication from a very young age, there are very limited availability and accessibility of education material for children. The aim of this study is to assess the impact of read-along videos of pictorial storybooks on children’s knowledge about rational use of medicines. This study compared pre and post knowledge scores in a nonrandomized, one-group pre-test-post-test experimental design. Pre-recorded read-along storytelling videos were used as intervention covering two topics on rational use of medicine -medicine storage and antibiotic resistance. The questionnaire and intervention videos were distributed using Google Forms to children aged six and seven in Malaysia *via* online social media platforms. 521 children completed the study. The mean baseline knowledge score for medication storage was 4.89 (SD = 1.12) pre-test and 5.44 (SD = 0.78) post-test while for antibiotic resistance the mean was 3.616 (SD = 1.340) pre-test and 4.820 (SD = 1.134) post-test. A Wilcoxon signed-rank test showed statistically significant changes on medication storage (Z = −10.21, *p* < 0.001) and antibiotic resistance (Z = −14.869, *p* < 0.001) related knowledge among children. Pictorial storybook read-along video interventions were shown to be effective in improving children’s knowledge on rational use of medicine. Education and awareness on the use of antibiotics should be prioritized.

## 1 Introduction

In 2007, the World Health Organization (WHO) released a report outlining global strategies to promote rational use of medicine which include policy coordination, providing unbiased information and promoting public education on medicines (World Health Organization, 2007). In response to this, Malaysia has launched a national health campaign known as “Know Your Medicine” which aimed to promote quality use of medicine through patient education (Pharmaceutical Services Division, 2022). Since then, various initiatives and materials have been taken to educate the public including training modules, printed and digital promotional materials, talks, media appearance in television, radio, newspapers, and social media platforms (Ahli Farmasi Rakan Ubat Anda, 2021; Duta Kenali Ubat Anda, 2022; Know Your Medicine Talk at, 2021; Know Your Medicines Ambassador, 2021; Hayin and Asyikin, 2019).

Medication literacy plays a significant role in determining treatment outcomes. The ability of patients to acquire, understand and use information about their medications affects their knowledge, skills, and confidence to manage their health conditions (Yadav et al., 2019). Several studies have showed important correlation between medication literacy, patient empowerment and improvement of self-management behaviors. Interactions between patient empowerment (PE) and communicative and critical health literacy (CCHL) at baseline were found to be significantly associated with 1-year global self-management behaviors. In a prospective study among Type 2 Diabetes Mellitus (T2DM) patients, PE was reported to improve self-management behaviors in patients with high CCHL but was less effective in patients with low CCHL (Wang et al., 2016). In addition, a meta-analysis by He et al. (He et al., 2017) showed that diabetes self-management education significantly reduced all-cause mortality risk in type 2 diabetes patients.

Medicines are regularly used to treat common acute illness in children. However, as the prevalence of chronic diseases such as asthma and diabetes showed increasing pattern among children, medication use in this group is expected to be more common (Al-Rubeaan, 2015; Sha et al., 2015). A cross sectional population-based study conducted in Saudi Arabia involving 23,523 participants showed high prevalence of diabetes in children and adolescents at approximately 10.84% (Al-Rubeaan, 2015). A qualitative study conducted among pediatric patients reported that majority of them managed their medications independently despite minimal knowledge on their medications. In terms of counselling experiences, both parents and pediatric patients were receptive for medication counselling particularly during prescription changes or initiation of new medication (Abraham et al., 2017). A study among adolescents in Taiwan presented that participants with lower medication knowledge, lower self-efficacy and lower medication literacy were more likely to engage in inappropriate self-medication (Lee et al., 2017).

In order to optimize the role of medication literacy and patient empowerment in health management, those two components should be aligned to individual’s cognitive, affective and behavioral abilities (Velasco et al., 2021). Childhood is a critical stage for development of fundamental cognitive, physical, and emotional processes. Intervention at early childhood will promote development of good health-related behaviors and ameliorate future health risks (Bröder et al., 2017). Although children are often considered too young to be responsible and independent of their medications, early medication education exposure will help in shaping their behavior and attitude toward medication later in life (Hampson et al., 2015). Study among pediatric patients highlighted the potential utilization of interactive and educational technologies to facilitate pharmacist’s counseling and educate children about the effective and safe use of medicines (Abraham et al., 2017).

Malaysia has taken the initiative to address these issues by producing medication-related modules for children. The modules are incorporated in the primary school health curricular under the rational use of medication syllabus. Education training modules on medication were also conducted by trained pharmacists through mass lecture, workshop, or small group work. Despite the efforts, the availability and accessibility to these children education materials are still very limited. In addition, content of the existing materials is insufficient for children use. For example, the topic on rational medication use comprised of only four pages in primary 1 (children aged 7) textbook (Azhar et al., 2016). In view of the lack of medication education materials for children, Aras Mega in collaboration with Pharmaceutical Services Program has produced a series of pictorial storybooks on medication use. The series were published in 2019 under the *Siri Kenali Ubat* (Know Your Medicine Series).

To date, there are limited studies assessing the impact of these materials on medication literacy among children. Therefore, in this study we aimed to assess the impact of read-along videos of pictorial storybooks on children’s knowledge about rational use of medicines. Methods and materials.

## 2 Methods

### 2.1 Study design

Following Fraenkel and Wallen (Fraenkel and Wallen, 1990), one-group pre-test-post-test experimental design was adopted for this study. Table 1 illustrates the one-group pre-test-post-test experimental design.

**TABLE 1:** One group Pre-test-Post-test Design.

Pre-test	Intervention	Post-test
Children to complete a questionnaire on knowledge about rational use of medication	Participants will watch the 2 volumes of *Siri Kenali Ubat* in pre-recorded read along storytelling videos	Children to complete a questionnaire on knowledge about rational use of medication

**FIGURE 1:**
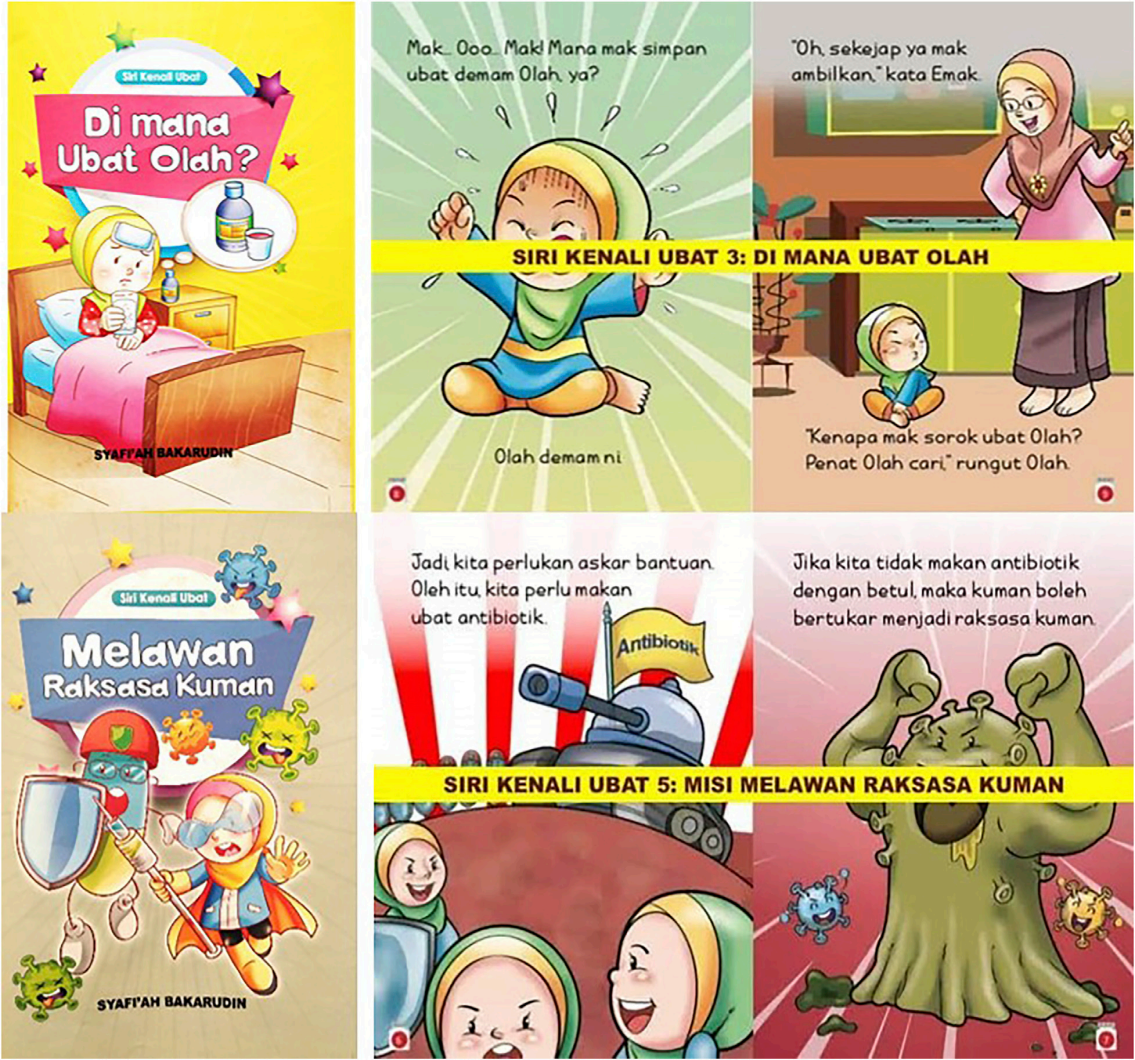
The two volumes of *Siri Kenali Ubat* book series.

*Siri Kenali Ubat* is a medication education material tailored for children, which portrays stories using characters named *Olah* who is a child, *Emak* who is *Olah*’s mother and *Uwan* who is *Olah*’s grandmother. One of the authors of this manuscript, SB, is the main author of these books. This series has five volumes. Only two volumes (Figure 1) were used in this study: 1) *Di Mana Ubat Olah?* (Where is Olah’s Medicine?), which emphasizes on proper storage of medicine by illustrating a story of *Olah* who’s have had a fever and is searching for medicine all around the house. 2) *Misi Melawan Raksasa Kuman* (Mission Against Monster Germ) explains about antibiotic resistance and the importance of taking antibiotic appropriately by illustrating a war between the body’s immune system army against bacteria. Using topics that are related to common medication concerns, the series come with features that fit children’s reading preferences such as big fonts, colorful and simplified facts in a narrative fiction together with captivating illustration and graphics.

Our initial plan was to have a face-to-face interaction with the school children at their school and allowing them to go through and read the books. However, during the study period, due the COVID-19 pandemic, all schools were closed to curb the infections. Alternatively, the research team decided to video-record one of the authors (SB) narrating the story to the children by flipping the pages one-by-one. Storytelling is a well-known practice that facilitates children’s language development and learning. It has been shown that read-aloud or read-along is effective in helping young children to develop lifelong literacy skills and behaviors (Malin, 2010).

### 2.2 Instrument

**FIGURE 2:**
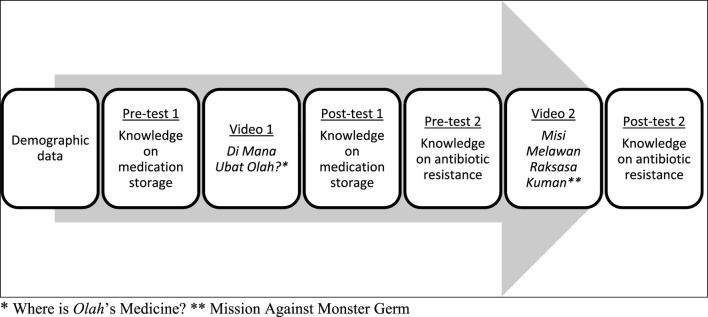
Flow of the survey.

Developing a survey questionnaire for children is challenging. However, after considering many factors (Bell, 2007) and discussion with research team member, a simple and structured questionnaire was developed in *Bahasa Melayu*, our national language, based on the topics covered in the two books. The flow of the survey is shown in Figure 2. Questionnaire was transformed into online format by using Google Form and the videos were attached in the link. Same questionnaire was used for both pre- and post-tests. A pilot test involving 5 to 10 children aged 6–7 years was conducted. Minor changes in terms and sentences were made based on the pilot test.

Parents’ and children’s consents were obtained prior to starting the survey. The survey instrument was administered and distributed through online social media platform such as WhatsApp, Facebook and Instagram.

Answers for pre- and post-tests were in the form of either “Yes”, “No” or “Don’t know”. A maximum score of six for each test is possible when all the questions are answered correctly. Correct answers will be given 1 mark while answers with “Don’t know” or wrong answers will be given a 0.

Each child watched the video with his/her parents. Parents could guide the participants by reading the questions and answer options. They were advised not to lead the participants to the correct answers and to encourage participants to answer based on their understanding.

Face and content validation by an expert panel consisting of academicians, pharmacists, and schoolteachers (*n* = 5) who have years of experience in related field was also conducted before the questionnaire was distributed to the participants. The knowledge section of the survey demonstrated good internal reliability and consistency with a Cronbach’s alpha value of 0.70.

### 2.3 Sample

Children aged 6–7 years old in 2021 across Malaysia represent the population of this study. Children who have been exposed to *Siri Kenali Ubat* books were excluded from this study and this was one of the screening questions in the questionnaire.

### 2.4 Ethical consideration

Ethical approval was obtained from the Universiti Teknologi MARA ethical committee [REC/05/2021 (MR329)].

#### 2.5 Data analysis

Level of knowledge was classified into three levels based on Bloom’s cutoff: high level (80–100%), moderate level (60–79%), and low level (<60%) (Bloom, 1956). Thus, a high-level score was 5–6 (80–100%), moderate level was 4 (60–79%), and low level was 0–3 (<60%) for medication storage and antibiotic resistance (Table 2). Whereas for total score on rational use of medicine, low level was a score of 0–7, moderate level was 8–9 and high level was 10–12.

**TABLE 2:** Classification of level of knowledge.

Level of knowledge	*Medication Storage/Antibiotic resistance*	*Rational use of medicine*
	Score (max = 6)	Total score (max = 12)
Low (<60%)	0–3	0–7
Moderate (60–79%)	4	8–9
High (80–100%)	5–6	10–12

Descriptive analysis was used to describe sample distribution and demographic data. The data was checked for meeting analysis assumptions and data entry together with statistical analysis was carried out using SPSS version 21.0 (SPSS Inc. Chicago, IL). Despite meeting the assumption of independent, the data has heterogeneous distribution between normal and non-normal distribution. Thus, non-parametric analysis was adopted for this study. Wilcoxon Sign-Ranked test was used to assess the differences of score before and after intervention within the same group. McNemar test was used to compare categorical data. Significance level was set at *α* < 0.05.

## 3 Results

### 3.1 Characteristics of the study population

During the 6 weeks of data collection from 12 July to 22 August 2021, 578 children participated in the study. Only 531 children met the inclusion criteria and 10 children were further excluded due to unavailability of consent from either the parents or children. Thus, 521 children were included for further analysis. Table 3 summarizes the demographic characteristics of participants in this study.

**TABLE 3:** Socio-demographic characteristic (*n* = 521).

Characteristics	Number of children (%)
Age (years)	
6	357 (68.5)
7	164 (31.5)
Gender	
Male	265 (50.9)
Female	256 (49.1)
Ethnicity	
Malay	502 (96.4)
Others	9 (1.7)
Chinese	5 (1.0)
Indian	5 (1.0)

### 3.2 Knowledge on rational use of medicine

The children’s baseline knowledge is generally high for medication storage and low for antibiotic resistance but was seen to be mixed in the total score as demonstrated in Table 4.

**TABLE 4:** Level of participants’ knowledge on medication storage, antibiotic resistance and rational use of medicine.

Level of knowledge	Pre-test, *n* (%)	Post-test, *n* (%)
Medication Storage		
Low	54 (10.4)	11 (2.1)
Moderate	98 (18.8)	55 (10.6)
High	369 (70.8)	455 (87.3)
Antibiotic Resistance		
Low	226 (43.4)	58 (11.1)
Moderate	156 (29.9)	141 (27.1)
High	139 (26.7)	322 (61.8)
Rational Use of Medicine (Total score of medication storage and antibiotic resistance)		
Low	154 (29.6)	29 (5.6)
Moderate	183 (35.1)	124 (23.8)
High	184 (35.3)	368 (70.6)

Approximately 10% of the children (*n* = 54) had a low level of knowledge, 18.8% (n = 98) a moderate and more than half (70.8%, *n* = 369) classified as having a high level of knowledge on medication storage pre-intervention. Post-intervention, only 2.1% (*n* = 11) had a low level of knowledge, 10.6% (*n* = 55) a moderate and more than three quarter (87.3%, *n* = 455) was classified as having a high level of knowledge. The mean baseline knowledge score for medication storage is 4.89 (SD = 1.12) for pre-test and 5.44 (SD = 0.78) for post-test. Inspection of the skewness, kurtosis and Kolmogorov-Smirnov statistics indicated that the assumption of normality for mean difference between pre and post-test was not supported. A Wilcoxon Signed Rank test (Table 5) showed that the 4 min story telling video using the book *Dimana Ubat Olah*? Elicit a statistically significant change on medication storage knowledge among children (Z = −10.21, *p* < 0.001).

**TABLE 5:** Result of analysis of the effect of *Dimana Ubat Olah?* And *Misi Melawan Raksasa Kuman* video on Children’s knowledge.

Topic (score range)	Mean score		Z^a^	*p*-value
	Pre-test	Post-test		
Medication storage (0–6)	4.889	5.441	−10.207	<0.001
Antibiotic resistance (0–6)	3.616	4.820	−14.869	<0.001
Rational use of medicine (0–12)	8.505	10.261	−15.261	<0.001

a Wilcoxon-signed ranks.

At baseline, 43% (*n* = 226) of the children were classified as having a low level of knowledge on antibiotic resistance and 30% (*n* = 156) had a moderate level of knowledge while only 27% (*n* = 139) was classified of having a high level of knowledge on antibiotic resistance (Table 4). After intervention, higher scores were seen. The percentage of children with a low level of knowledge was 11.1% (*n* = 58), a moderate level of knowledge was 27% (*n* = 141) whereas 62% had a high level of knowledge (*n* = 322). The mean baseline knowledge score for medication storage was 3.616 (SD = 1.340) and 4.820 (SD = 1.134) for post-test. As assumption of normality for mean difference between pre and post-test was not supported, non-parametric test was used. A Wilcoxon signed-rank test showed that the 5 min story telling video using the book *Misi Melawan Raksasa Kuman* resulted in a statistically significant change on antibiotic resistance topic knowledge among children (Z = −14.869, *p* < 0.001) (Table 5).

The improvements were also evident and significant in each question pre- and post-intervention for both topics. Table 6 summarized the results for each given question. For the question on “Medicine can be kept on dining table”, at baseline 81% (*n* = 422) of the children answered correctly. After intervention, 15.2% (*n* = 79) changed their answers to correct answer giving a total of 94%. Similar changes were seen in questions on “Medicine can be kept near the cooking hob” (97.1 vs. 99.8%), “Medicine should be kept away from children” (93.1 vs. 97.7%), “Medicine should be kept away from hot temperature and direct sunlight” (80.4 vs. 89.8%), “Medicine should be kept in a wet and damp area” (85.4 vs. 92.9%) and “All medicine should be kept in refrigerator” (51.8 vs. 69.9%).

**TABLE 6:** Children knowledge changes based on questions.

Question	Correct answer (%)		^a^ *p*-value
	Pre-test	Post-test	
Medication storage			
1. Medicine can be kept on dining table	81.0	94.0	<0.001
2. Medicine can be kept near the cooking hob	97.1	99.8	<0.001
3. Medicine should be kept away from children	93.1	97.7	<0.001
4. Medicine should be kept away from hot temperature and direct sunlight	80.4	89.8	<0.001
5. Medicine should be kept in a wet and damp area	85.4	92.9	<0.001
6. All medicine should be kept in refrigerator	51.8	69.9	<0.001
Antibiotic Resistance			
1. Antibiotic helps our body to kill bacteria	89.3	98.5	<0.001
2. Antibiotic also helps our body to kill virus	19.2	51.6	<0.001
3. Antibiotic should be finished even the patient has recovered	77.9	84.6	<0.001
4. If antibiotic is not finished, bacteria that was not killed by the antibiotic will turn to a stronger monster	58.7	88.5	<0.001
5. Germ monster is dangerous and may cause death	73.3	90.2	<0.001
6. All disease requires antibiotic treatment	43.2	68.5	<0.001

a McNemar test.

In addition, for the question on “Antibiotic helps our body to kill bacteria”, at baseline 89.3% (*n* = 465) of the children answered correctly (Table 6). After intervention, 10.2% (*n* = 53) changed their answers to the correct answer (*n* = 513, 98.5%). Following that, at baseline, 81% (*n* = 421) children believed that antibiotic helps to kill virus and post-intervention more children provided the correct answer (19 vs. 52%). Similarly, at baseline 57% (*n* = 296) children believed that “All disease requires antibiotic treatment” and this perception changed after intervention where more children provided the correct answer (43.2 vs. 68.5%).

## 4 Discussion

Video storytelling using pictorial storybooks addressing medication storage and antibiotic resistance were found to result in a significant improvement in children’s knowledge. Various studies using storytelling methods found to have improved children’s vocabulary, language, mathematical reasonings and scientific concepts (Hassinger-Das et al., 2015; Kalogiannakis et al., 2018; Picton and Clark, 2019). However, to date no study evaluated the use of storybooks to improve knowledge on rational use of medication among young children.

In a similar approach, effectiveness of cartoon video on knowledge and oral hygiene among students with hearing disabilities revealed that the cartoon was able to enhance knowledge even in students with hearing impairment. The study found that mean score for children’s knowledge on oral health increased from 7.73 (SD ± 0.38) to 10.75 (SD ± 0.42) post 1 day and 14.23 (SD ± 0.38) post 1 week respectively (Yanti et al., 2017). Additionally, a non-randomized quasi-experimental pre-test and post-test study (Nurcahyani and Padmawati, 2019) investigated on early childhood education and reproductive health among children aged five to six. This study utilized stop-motion videos as an intervention and found substantial knowledge improvement in children’s knowledge scores after intervention. In our study, although the videos were not animated compared to the two studies mentioned above, the read-along video could mimic actual reading activity in-person and the results of significant improvement in knowledge authenticate the approach. Having said that, converting the *Siri Kenali Ubat* book series into animation characters and videos could be a promising field to explore in future.

A common educational intervention such as counselling has been utilized to improve knowledge on medications across different patient populations. A multisite, stepped-wedge trial in Colorado, United States which investigated the effectiveness of counselling intervention among caregivers of youths aged 10–17 years, reported a 2-fold improvement in medication storage after the intervention (Miller et al., 2020). Another study (Gregorian et al., 2020) also suggested that when compared to individuals who did not receive any educational intervention, those who did were more likely to keep their medications in a secured location. Whilst in children, the combination of verbal or written counselling with pictograms were found to be more effective in promoting proper use of medicines (Sletvold et al., 2020). This implies that the use of pictures and illustrations while educating children on rational use of medicines could amplify the transfer of knowledge.

In the United States, a 2018 National Household Medication Survey revealed that 76.7% households inappropriately stored at least one medication and 34.7% stored medication on counter tops where children could easily access them (Funk et al., 2021). In Brazil, a study revealed that households with insufficient storage conditions frequently kept medications within the reach of children (Martins et al., 2017). Majority users of high-risk medication such as opioid pain relievers were also found to have kept the medication in unsafe manner without lock or latched storage despite having children at home (McDonald et al., 2017). Our study found that at baseline, 93.1% of children agreed that medicines should be kept away from them and the percentage increased to 97.7% after the intervention. Although the children’s knowledge level looks promising, it is important to note that most acute drug poisoning cases in children that result in hospitalization are due to unintentional and accidental ingestion (Bell et al., 2018; Matalová et al., 2019). Thus, we cannot emphasize enough on educating children and their parents on the importance of safe medication storage.

In this study, compared to medication storage, children seem to have a lower level of knowledge on antibiotics and antibiotic resistance at baseline. However, this improved after the intervention. 81 and 57% of children in this study believed that “antibiotics are used to kill virus” and “all diseases require antibiotics”, respectively. Although there was an increase in the percentage of correct answers after intervention, only half of the children were able to correctly identify that antibiotics are not able to kill the viruses. A study in Ghana investigating the effectiveness of storytelling and picture drawing among children revealed that 81% children believed antibiotics will cure most coughs and colds (Appiah et al., 2021). This percentage reduced to 63% after intervention. Despite intervention to educate children, they seem to have a lack of understanding of the purpose of antibiotics. Children rely on parents for right information, however, even parents are misinformed regarding the use of antibiotics. In a study conducted in China, 79% of parents thought that antibiotics could cure viral infections (Yu et al., 2014).

Antibiotic resistance has been a topic of interest for many years and yet there is an incomplete understanding of and misperceptions about it (McCullough et al., 2016). Children are not exceptional (Deo et al., 2018). Misuse and overuse of antibiotics are one of the major reasons leading to antibiotic resistance (World Health Organization (WHO), 2017). Thus, it is imperative to create awareness on safer use of antibiotics. Children’s involvement as active participants in health care efforts is usually underappreciated. Little is known about how to create instructional resources for children that will allow them to be the “changing agents” in their communities. Therefore, this study serves as a good example on establishing engagement with children in providing them a basic knowledge on antibiotic resistance to empower rational use of antibiotic among the children and eventually the community. However, improvement in knowledge regarding antibiotics may need a long-term effort and education materials should include basic concepts of antibiotics, the appropriate indications and administration, and the potential hazards of antibiotics (McCullough et al., 2016).

### 4.1 Limitation and recommendation

We acknowledge several limitations in this study. There was a large demographic disparity which included a majority of participants from one ethnicity. This is mainly due to the use of convenience sampling method. In a multi-ethnic country, the findings from this study may not represent the whole Malaysian population. We were able to cover only two volumes from the *Siri Kenali Ubat* series instead of all five due to a concern on children’s attention time span. Future studies may explore other sampling strategies to ensure the sample to be more representative of entire Malaysian children population and evaluate the other volumes in the series.

In view of COVID-19 pandemic restriction, the schools were closed for physical operation. A face-to-face read along session was impossible and to adapt to the situation, read-along videos were developed. However, future research could explore other educational approaches such as physical story telling sessions or interactive activities together with some hands-on activities.

Another limitation that might be due to the cultural specificity of the population, is the fact that children already had a high level of knowledge on the topics investigated and we did not collect data on children’s medical illness and whether they are prescribed with long-term medication. Additionally, there may be a ceiling effect on learning as the survey was done in a close proximity to the videos. Thus, more studies will need to be conducted to find to what degree the positive impact found in this study will replicate for groups of children with low baseline knowledge levels and at different time span.

## 5 Conclusion

Pictorial storybook read-along video interventions using *Siri Kenali Ubat* series were shown to be effective in improving children’s knowledge on rational use of medicine on the topic of medication storage and antibiotic resistance. It is imperative to prioritize educating children on antibiotic use and resistance. Future studies should develop and assess children’s specific medication education materials in various topics to empower our future generation to be more aware and knowledgeable in rational drug use.

## Data Availability

The raw data supporting the conclusions of this article will be made available by the authors, without undue reservation.
